# EEG-based neonatal seizure detection with Support Vector Machines

**DOI:** 10.1016/j.clinph.2010.06.034

**Published:** 2011-03

**Authors:** A. Temko, E. Thomas, W. Marnane, G. Lightbody, G. Boylan

**Affiliations:** a Neonatal Brain Research Group, University College Cork, Ireland; b Department of Electrical and Electronic Engineering, University College Cork, Ireland; c Department of Paediatrics and Child Health, University College Cork, Ireland

**Keywords:** Neonatal EEG, Automated seizure detection, Machine learning, Support Vector Machines

## Abstract

**Objective:**

The study presents a multi-channel patient-independent neonatal seizure detection system based on the Support Vector Machine (SVM) classifier.

**Methods:**

A machine learning algorithm (SVM) is used as a classifier to discriminate between seizure and non-seizure EEG epochs. Two post-processing steps are proposed to increase both the temporal precision and the robustness of the system. The resulting system is validated on a large clinical dataset of 267 h of EEG data from 17 full-term newborns with seizures.

**Results:**

The performance of the system using event-based metrics is reported. The system showed the best up-to-date performance of a neonatal seizure detection system. The system was able to achieve an average good detection rate of ∼89% with one false seizure detection per hour, ∼96% with two false detections per hour, or ∼100% with four false detections per hour. An analysis of errors revealed sources of misclassification in terms of both missed seizures and false detections.

**Conclusions:**

The results obtained with the proposed SVM-based seizure detection system allow for its practical application in neonatal intensive care units.

**Significance:**

The proposed SVM-based seizure detection system can greatly assist clinical staff, in a neonatal intensive care unit, to interpret the EEG. The system allows control of the final decision by choosing different confidence levels which makes it flexible for clinical needs. The obtained results may provide a reference for future seizure detection systems.

## 1 Introduction

Seizures in newborn babies are commonly caused by problems such as lack of oxygen, haemorrhage, meningitis, infection and stroke. The incidence of clinically apparent neonatal seizures is generally reported as around 3 per 1000 and under certain circumstances, such as in very preterm babies, 50 per 1000 (Rennie and Boylan, 2007). In reality, these values are probably underestimates as approximately one-third of all seizures are clinically visible and only around 1/10 are actually documented (Murray et al., 2008). Failure to detect seizures and the resulting lack of treatment can result in brain damage and in severe cases, death.

Seizures are missed because they are very difficult to detect clinically; unlike older children and adults, babies do not always exhibit obvious clinical signs during seizures. EEG is the only available method to detect all seizures in babies. Most neonatal intensive care units (NICUs) lack the expertise required to interpret multi-channel EEG results, particularly on a 24/7 basis. Instead they often rely on amplitude integrated EEG systems (aEEG) that employ one or two channels of EEG and interpretation of a compressed and highly filtered output. Seizure detection using aEEG systems is heavily dependent on the experience of the user, the frequency and amplitude of seizures and their location and duration. It is not surprising therefore that seizure detection rates ranging from 12% to 76% have been reported using aEEG (Bourez-Swart et al., 2009; Klebermass et al., 2001; Rennie et al., 2004; Shah et al., 2008; Toet et al., 2002; van Rooij et al., 2009). We and others have reported the discrepancy that exists between seizure number and actual seizure burden, i.e. the total amount of time the baby spends in seizure (Murray et al., 2008). Therefore for our seizure detection work we have been keen to express seizure burden as well as the number of seizure events when reporting system performance (Temko et al., 2010).

A system that could automatically detect and annotate seizures on the neonatal EEG would be extremely useful to clinicians in the neonatal intensive care unit. Although a number of methods and algorithms have been proposed previously in an attempt to automatically detect neonatal seizures, to date their transition to clinical use has been limited due to poor performance.

Numerous approaches have been proposed to identify and quantify the increase in periodicity of the EEG seen during neonatal seizures. Spectral analysis (Gotman et al., 1997), autocorrelation based metrics (Liu et al., 1992) and singular value decomposition (Celka and Colditz, 2002) were tested in an independent study (Faul et al., 2005a), with results proving unsatisfactory for clinical implementation.

A method to mimic a human observer using a detector designed to identify spike-train like seizures and a second detector looking for oscillatory seizures has recently been proposed by Deburchgraeve et al. (2008). Features related to amplitude, period and linear correlation of wave sequences of the EEG to produce elementary decisions are exploited in (Navakatikyan et al., 2006).

Instead of using a set of heuristic rules and thresholds, several approaches utilise a classifier – a data-driven set of thresholds automatically trained on the data. A system based on a multilayer perceptron to classify neonatal EEG into one of six background states or two seizure states has been proposed by one group (Aarabi et al., 2007). Our group has previously described neonatal seizure detectors that have been built on the basis of linear, quadratic and regularised discriminants (Greene et al., 2008a).

Recent work on statistical machine learning has shown the advantages of discriminative classifiers such as Support Vector Machines (SVM) in a range of applications (Vapnik, 1982; Scholkopf and Smola, 2002), including seizure detection. The SVM is a discriminative model classification technique that mainly relies on two assumptions. First, transforming data into a high-dimensional space may convert complex classification problems (with complex decision surfaces) into simpler problems that can use linear discriminant functions. Second, SVMs utilise only those training patterns that are near the decision surface assuming they provide the most useful information for classification. SVM was initially developed as a binary classifier and thus it is very well suited to binary classification problems such as seizure detection. A patient-dependent neonatal seizure detection system based on a continuously-retrained SVM has been proposed but has only been tested on a 12-h recording from a single newborn (Runarsson and Sigurdsson, 2005). A one-class SVM methodology has also been used for seizure detection from intracranial EEG in adults (Gardner et al., 2006).

In this study, a multi-channel patient-independent neonatal seizure detection system was designed, based on an SVM classifier and retrospectively evaluated on a large clinical dataset (Temko et al., 2009). By varying the level of confidence of the system decisions, the curve of performance is reported.

## 2 Methods

### 2.1 Dataset

In this study we used a dataset of recordings from 17 newborns in the Neonatal Intensive Care Unit (NICU) of Cork University Maternity Hospital, Cork, Ireland recorded over a 2 year period. During this period, 55 full-term babies with Hypoxic Ischaemic Encephalopathy (HIE) were recruited and EEGs were recorded for up to 72 h; 17 had seizures. Further details regarding the dataset can be found in Table 1. A Viasys NicOne video EEG machine was used to record multi-channel EEG at 256 Hz using the 10–20 system of electrode placement modified for neonates. The following eight bipolar EEG channels are used in this study: F4–C4, C4–O2, F3–C3, C3–O1, T4–C4, C4–Cz, Cz–C3 and C3–T3. The combined length of the recordings totals 267.9 h (mean duration per patient is 15.76 h) and contains 705 seizures which range from less than 1 min to more than 10 min in duration (mean seizure duration is 3.89 min). The dataset contained a wide variety of seizure types including both electrographic-only and electro-clinical seizures of focal, multi-focal and generalized types. Previous study (Murray et al., 2008) examined 526 electrographic seizures from nine seizure patients in our dataset and showed that 34% had clinical manifestations evident on the simultaneous video recording. All seizures were annotated independently by two experienced neonatal electroencephalographers using video EEG. The continuous EEG recordings were not edited to remove the large variety of artifacts and poorly conditioned signals commonly encountered in the real-world NICU environment. Therefore this dataset is truly representative of the real-life situation in the NICU and it allows the most robust estimate of the algorithm’s performance.

Seizure is not always present in all EEG channels. Therefore in order to train a system for detection of neonatal seizures, it is necessary to know the channels involved in the seizure. For this purpose, a 2-min file with seizure activity was also created for each patient from the above-described dataset. The individual channel annotations were then produced for each file. This seizure data was then used for training the classifier model as explained in Section 2.2.4.

### 2.2 SVM-based seizure detection system

#### 2.2.1 System overview

**Fig. 1:**
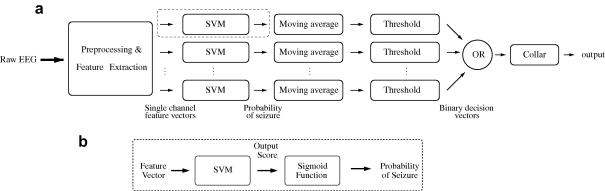
(a) Architecture of the SVM-based seizure detection system. Various stages of the algorithm such as feature extraction, classification and post-processing are schematically shown. (b) The detailed structure of SVM classification which includes converting raw SVM outputs to probabilistic values.

The outline of the system is shown in Fig. 1a and b. First, the signal from each EEG channel is separately pre-processed and segmented into epochs. A set of features is extracted from each EEG epoch. The feature vectors are then fed to the SVM classifier where a probability of a seizure is obtained for each EEG epoch. These probabilities are smoothed with a central moving average filter and transformed into binary decisions (Whittaker and Robinson, 1967). The single channel binary decisions are then combined into a multi-channel binary decision. A final post-processing step is a collar operation which consists of expanding all seizure (positive decisions in our case) events forward and backward in time.

#### 2.2.2 Preprocessing

The EEG is down-sampled from 256 to 32 Hz with an anti-aliasing filter set at 12.8 Hz. It has been shown that frequencies of neonatal EEG seizures range between 0.5 and 13 Hz and the dominant frequencies of seizures vary between 0.5 and 6 Hz (Kitayama et al., 2003). The EEG is split into 8s epochs with 50% overlap between epochs. The most recent recommendations by the International Federation of Clinical Neurophysiology (IFCN) (De Weerd et al., 1999) suggest that 5 s is the minimum seizure duration if the background EEG is normal and 10 s if the background EEG is abnormal. A window length of 8 s was chosen given that most babies with HIE and seizures have an abnormal background EEG. This window length would also prevent short duration seizure-like events (e.g. brief intermittent rhythmic discharges) being incorrectly detected as seizure events.

#### 2.2.3 Feature extraction

The features which have been shown to be useful for neonatal seizure detection in a number of papers are extracted for each epoch (Gotman et al., 1997; Aarabi et al., 2007; Faul et al., 2005b; Greene et al., 2008a). The frequency domain features are extracted from the power spectrum density (PSD) of each epoch which is obtained using a 256 point Fast Fourier Transform (FFT). A number of time-domain features are extracted directly from the epoch of EEG along with the first and second derivative of the EEG denoted by Δ and ΔΔ, respectively. Based on an analysis of features, several features based on information theory were also chosen (Faul et al., 2005b). The exact formulae and a discussion on the importance of each feature is described elsewhere (Greene et al., 2008b). The features are listed in Table 2.

In total, 55 features are extracted. Despite the fact that some features may be redundant, preliminary experiments confirmed that the SVM was not very sensitive to their presence which has also been shown in the literature (Weston et al., 2000). Indeed, it has been reported that omitting good features may be more detrimental for SVMs than including bad ones (Guyon et al., 2008). Initial tests with the recursive feature elimination technique (Guyon et al., 2002) showed that the best results were obtained using all of the extracted features together, and no other feature selection techniques tested gave significantly better results. In addition, since the system that uses all of the features still functions faster than real-time, and no particular low-power ambulatory application is intended, all 55 features are used throughout the work. The feature combination/ranking/selection results are therefore not presented in this paper but can be found in our separate study (Temko et al., in press).

#### 2.2.4 SVM classifier

The extracted feature vectors are classified using a SVM classifier. The SVM is a discriminative model classification technique that mainly relies on two assumptions (Scholkopf and Smola, 2002). First, transforming data into a high-dimensional space may convert complex classification problems (with complex decision surfaces) into simpler problems that can use linear discriminant functions. Second, SVMs utilise only those training patterns that are near the decision surface assuming they provide the most useful information for classification. More details on the construction of the SVM classifier are given in Appendix A.

Classification consists of two steps – training and testing. The leave-one-out cross-validation method is used to assess the performance of the system for patient-independent seizure detection. This way, all but one patients’ data is used for training and the remaining patient’s data is used for testing. This procedure is repeated until each patient has been a test subject and the mean result is reported. The leave-one-out method is known to be an almost unbiased estimation of the true generalization error; that is, the performance reported with the leave-one-out method is the most similar to the performance this system would show on an unseen test dataset of infinite length once it is trained on all available data (Vapnik, 1982).

In the training stage, seizure and non-seizure epochs are labelled 1 and −1, respectively, for each channel. For the seizure class, the training dataset consists of 2 min of EEG per patient for which individual channel annotations are available, which sum up to *M* * 2 min per patient for seizures involved in *M* channels. For example, if a training dataset consists of 16 patients for which 2 min of seizure are transcribed and on average four channels are involved in every seizure, then for an epoch length of 8 s with an overlap of 4 s, the seizure class of training data will consist of 16 patients * (120 s/4 s) * 4 channels = 1920 epochs. It may be more or less depending on the number of channels involved in every seizure for every patient. For the non-seizure class, 10,000 epochs of the non-seizure data are randomly selected from all channels of all patients in training dataset. The features extracted from each epoch are then fed to train one SVM classifier. The training data for the SVM classifier are first normalized anisotropically by subtracting the mean and dividing by the standard deviation to assure commensurability of various features. The obtained normalizing template is then applied to the testing data. In this work, a Gaussian kernel is employed. For model selection (to search for optimal Gaussian kernel parameter and generalization parameters), fivefold cross-validation on training data is used. Once the optimal pair of parameters is found, it is used to train the final model on all the training data.

In the testing stage, the obtained classifier is applied separately to each channel of the testing data and the decisions are post-processed and fused as described below.

#### 2.2.5 Post-processing

**Fig. 2:**
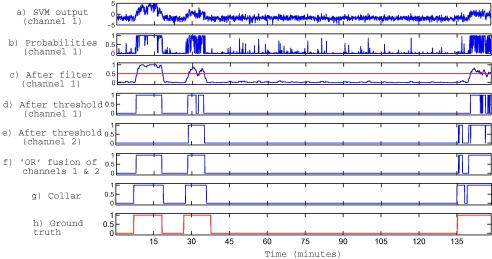
Effects of the post-processing scheme. (a) The raw output of the SVM classifier. (b) The output converted to a probability via a sigmoid function. (c) The smoothed output after a 15-tap moving average filter is applied. (d) The binary decisions resulting from applying a threshold of 0.5 to the filtered probabilities of seizure. (d) All seizures are detected using binary decisions however total seizure burden is underestimated. (e) The binary decisions from another EEG channel where one seizure is missed. (f) The binary decisions after two channels which are shown in plot (d) and (e) are fused. (g) The final binary decisions after the collar operation, which increases the duration of all positive decisions. (h) The ground truth, where 1 indicates seizure.

Post-processing is a technique which is applied to the classifier outputs. As can be seen in Fig. 1a and b the post-processing scheme consists of a conversion of the SVM outputs to probabilistic values, filtering, applying a threshold, and multi-channel decision-making. The effects of post-processing can be seen in the example shown in Fig. 2.

Every epoch is represented by a feature vector in each channel. The output of the SVM classifier is computed for each epoch (Fig. 2a). These outputs are then converted to posterior probabilities (Fig. 2b) using a sigmoid function:

(1) $$P(\left(y=1\right|f)=\frac{1}{1+\exp(\mathrm{Af}+B)}$$

where *f* is the distance to the separating hyperplane, i.e. the output of the SVM classifier, *A* and *B* are the parameters of the sigmoid function estimated on the training dataset using the method described by Platt (1999). For unbalanced problems, such as seizure detection, decisions made with a threshold given by the sigmoid function were shown to be significantly better than those obtained with the original threshold of zero applied to the distances *f* (Platt, 1999). Results in our study confirmed this. Additionally, the conversion to probabilistic values facilitates the choice of the desired operating point as the threshold has to be chosen from the bounded interval [0 1].

A central linear moving average filter (MAF) is applied to the time sequence of probabilities in each channel (Fig. 2c). It is defined as:

(2) $$y[i]=\frac{1}{2N+1}\overset{N}{\underset{j=-N}{\sum }}x[i+j]$$

where *x*[] is the input signal, *y*[] is the output (filtered) signal, 2*N *+ 1 is the order of the filter (the number of points used in the moving average). Essentially, the moving average filter is a convolution of the input signal with a rectangular pulse of unit area. It has been noted in Smith (1997) that the moving average filter is an exceptionally good smoothing filter. Moreover, this simple filter is an optimal filter for the common task of reducing random noise, while keeping the sharpest step response.

The averaged value is then compared to a threshold from the interval [0 1] (e.g. 0.5 for equal confidence/priors for the seizure and non-seizure classes as shown in Fig. 2c). To obtain curves of performance metrics the threshold can be gradually varied from 0 to 1. After comparison, binary decisions are taken per channel (Fig. 2d): 1 – seizure; 0 – non-seizure.

Then the procedure that is used in marking the data is employed: if there is a seizure in at least one channel, the whole epoch is marked as a seizure, otherwise it is denoted as a non-seizure. It corresponds to a logical ‘OR’ operator applied to the binary outputs from the channels. This process is shown in Fig. 2f as an example in which the binary decision from two channels (Fig. 2d and e) are fused with the ‘OR’ operator. Alternatively, the same post-processing effect would be achieved if the “MAX” operator is applied to the outputs of the MAF over channels and then the resulting maximum value is compared to the decision threshold.

The ‘collar’ technique used in speech processing applications to prevent cutting off the beginning and ending of words (Huang and Harper, 2005; Boakye, 2008) is then applied here. Every seizure decision is extended from either side (Fig. 2g) to compensate for possible difficulties in detecting pre-seizure and post-seizure parts.

### 2.3 Performance measurements

The event-based metrics which are thought to reflect the performance of a system for a specific application are used throughout the work. The subsequent decisions of the same class are joined to create an event. There are two scores defined. Good detection rate (GDR) is defined as the percentage of seizure events correctly identified by the system as labelled by an expert in neonatal EEG. If a seizure was detected any time between the start and end of a labelled seizure this was considered a good detection (Gotman et al., 1997). The other score is the number of false detections per hour (FD/h) calculated as the number of predicted seizure events in 1 h that have no overlap with actual reference seizures. The curve of variation of GDR with FD/h is reported in our study. To the best of our knowledge this has not been reported previously.

Additionally, an analysis of errors is performed in this work in order to gain further insight into the performance of the system. Specifically, missed seizures were analysed by grouping seizure events into four subsets according to their duration. Also every false alarm obtained by setting the system to a specific operating point was analysed to determine the nature of the EEG patterns that resulted in false detections. The false detections were visually grouped into three classes: artifact-free background activity, artifact contaminated EEG, and seizure-like activity.

## 3 Results

**Fig. 3:**
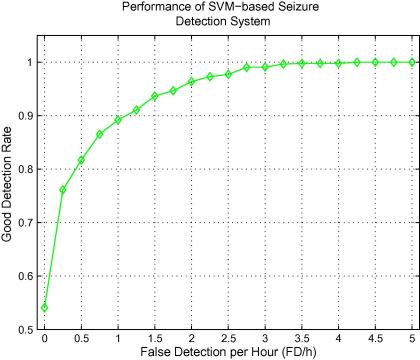
The curve of variation of the GDR against FD/h.

The overall performance of our system is seen in Fig. 3. The system can correctly detect ∼89% of seizure events with a cost of 1 FD/h, ∼96% with a cost of 2 FD/h, or ∼100% with a cost of 4 FD/h. Additionally, the system is able to detect more than 50% of seizures without a single false detection. The best performance was achieved with MAF = 15 and collar = 40 s and the curve of variation of GDR with FD/h is shown in Fig. 3. Table 3 shows the GDR at 0.5 FD/h and at 1 FD/h for each experiment in the leave-one-out performance assessment method. A significant improvement is achieved for GDR when changing the operating point from 0.5 FD/h to 1 FD/h. In 4 of 17 patients shown in Table 3 the classifier achieves a GDR of 100% at 0.5 FD/h. This can be increased to six patients with a false detection rate of 1 FD/h. For patients 1, 2, 7, 10 which are highlighted in Table 3, the GDRs are lower. Unlike other patients, the GDR for patients 1, 2, and 10 do not increase even when 1 FD/h is allowed. In fact, according to Table 1, the amount of time when patients 1, 2, 7 are having a seizure is less than 2% of total time.

### 3.1 Missed seizures

**Fig. 4:**
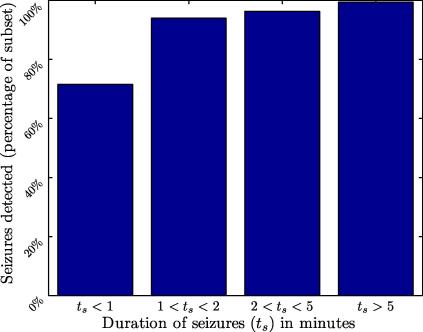
Detection of seizures of different duration by the SVM-based seizure detection system at 1 FD/h. The number of seizures in each time category is 72, 182, 240, and 197.

In order to gain further insight into the performance of the system, missed seizures were analysed. Seizure events were grouped into four subsets according to the duration of the seizure event and the percentage of seizures detected is shown in Fig. 4 for each subset. As can be seen from Fig. 4, seizures of short duration – in particular those lasting less than 1 min – are the most difficult to detect. It should be noted that the duration of neonatal seizures is typically longer than 1 min (Clancy and Legido, 1987). However, for patients 1, 4, 6, and 7 a number of seizures are shorter than 1 min. It can also be seen that the system detects almost all seizures of duration longer than 5 min, an important subset of seizures to detect accurately. Additionally, the system achieves a GDR of over 90% for all seizures of duration above 2 min. The relatively low GDR scores (∼70%) for shorter seizures can be attributed in part to the moving average filter length. This is due to background EEG reducing the mean probability of seizure score for seizures lasting less than the length of the moving average filter. The moving average filter is 15 epochs in length, corresponding to 64 s, as this length was found to maximise the performance. It is attributable to the lower number of short duration seizures in the database (144/705).

It should be noted that in this study only full-term babies with HIE were included and generally seizures in this group are over 1 min in duration. Some seizures in neonates can be of short duration particularly in preterm babies. In clinical practice, a single short duration seizure in a neonate lasting 10 s or a little longer would rarely be treated.

**Fig. 5:**
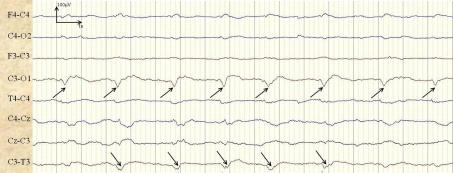
Example of a missed seizure. The seizure is indicated with arrows in channels C3–O1 and C3–T3.

An example of a longer seizure, missed by the presented automated neonatal seizure detection system, from patient 16 is shown in Fig. 5. The seizure lasted 3.3 min and 17 s of its central section are shown.

### 3.2 False detections

**Fig. 6:**
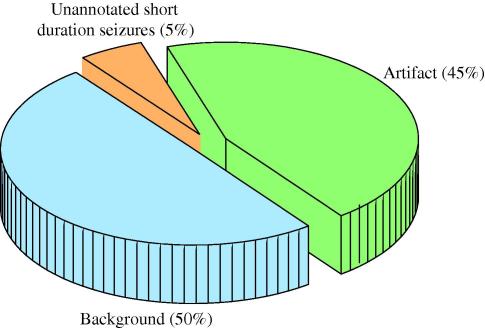
Most frequent sources of false detections produced by the SVM-based seizure detection system at 1 FD/h.

The EEG was reviewed for each of the 277 false alarms obtained by setting the system to 1 FD/h operating point (∼1.03 FD/h actual) to determine the nature of the EEG patterns that resulted in false detections. The false detections were visually grouped into three classes as shown in Fig. 6: artifact-free background activity, artifact contaminated EEG, and seizure-like activity.

From Fig. 6, it can be seen that false alarms are mainly due to both background activity and artifact contamination of the EEG record. It should be taken into account that no part in the current system is responsible for artifact rejection or removal. In fact, artifacts are only implicitly tackled at the classification stage when modelled within the non-seizure class. Therefore, the current actual errors which the system is truly responsible for belong only to the first group – background activity. However, the amount of errors caused by artifacts, reported in Fig. 6, indicates how significant a potential impact of a proper artifact rejection classifier running in parallel or preceding the seizure detector could be.

**Fig. 7:**
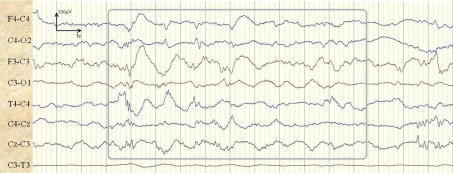
Example of background activity (trace-alternant) incorrectly classified as a seizure in channel F3–C3.

Some false alarms were seen during periods of usual background activity. Delta activity was found to trigger false alarms when the background patterns became more rhythmic or sharp. An example of a background pattern incorrectly classified as a seizure is shown in Fig. 7. A false detection of 1.5 min duration is produced in channel F3–C3 of patient 4. Fig. 7 shows 17 s of the central section of the incorrectly detected seizure.

**Fig. 8:**
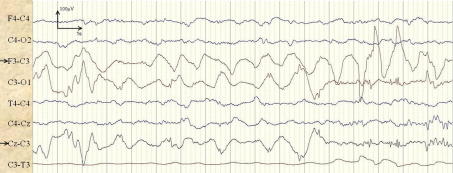
Example of an electrode-disconnect artifact after filtering incorrectly classified as a seizure in channels F3–C3 and Cz–C3.

The most prevalent artifacts causing false detections were electrode-disconnect, respiration and high amplitude activity caused by movement or handling of the patient. Electrode detachment was found to be the most predominant cause of false detections among artifacts. An example of electrode detachment artifact is shown in Fig. 8 where the electrode C3 is detached for patient 4. A seizure is incorrectly classified for duration of 1.75 min in channels F3–C3 and Cz–C3. In fact, Fig. 8 shows 17 s of the artifact which lasted over 3 h causing more than 10 separated false detections. This artifact is usually characterised by a large 50 Hz component as the electrode becomes contaminated with electrical noise from the environment. Although of course the 50 Hz component is removed via notch filtering; other environmental signals of lower frequency are present and preserved in the recording.

**Fig. 9:**
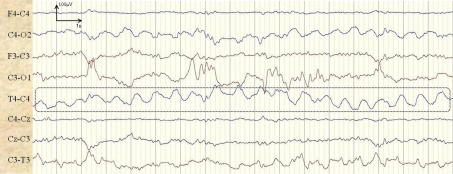
Example of a respiratory artifact incorrectly classified as a seizure in channel T4–C4.

An example of the respiration artifact is shown in Fig. 9. As can be seen it is repetitive in nature and thus is a cause of false alarms. Fig. 9 shows 17 s of the respiration artifact from patient 12 lasting over 6 min which was incorrectly classified as a seizure in channel T4–C4 for duration of 3.5 min.

**Fig. 10:**
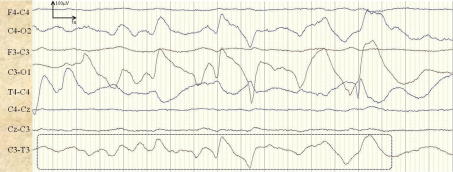
Example of a “being handled” artifact incorrectly classified as a seizure in channel C3–T3.

An example of the movement artifact from patient 12 which causes high amplitude patterns in the EEG is presented in Fig. 10. The artifact lasted 2 min and caused a false detection of the same duration in channel C3–T3.

It was found that approximately 5% of false detections occurred from short runs of seizure activity (10–15 s) that had not been annotated as seizures by the neurophysiologists. In all cases, these short duration seizures occurred during periods of status epilepticus or frequent seizures.

**Fig. 11:**
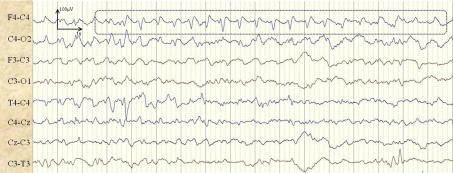
Example of a short duration seizure that was not annotated by neurophysiologists but detected with high confidence by the algorithm in channel F4–C4.

When we went back to the neurophysiologists they agreed that these should be labelled as seizures but felt that they were not significant enough to warrant annotation during the first assessment in view of the surrounding longer seizures (Fig. 11). However it is reassuring to note that the algorithm correctly identified these very short segments. It should be noted however that these new events would only increase the total number of annotated seizures by 2%. Clearly, the algorithm considered them to be sufficiently near in character to true seizures to highlight them. Neonatal seizures can often be difficult to distinguish from the background activity and, when multiple seizures are present, it is often difficult to clearly describe the end of one seizure and the beginning of another. Computerised detection methods may be able to do this better than the human observer. To date there have not been any studies of inter-observer agreement in neonatal seizure detection.

The integration of the feedback outlined in the analysis of errors to further improve the robustness and performance of the neonatal seizure detector is the scope of the future work. It is not trivial to figure out why the algorithm responded to certain characteristics of the signal: whether it happened because certain EEG activity was underrepresented in the training data or whether there is a certain feature (s) which considered this activity close enough to seizure and produced a ‘seizure’ response to this activity. For these reasons, we would like to avoid coming up with simple, untested and thus unreliable solutions to the identified problems but instead would rather perform a more thorough investigation.

## 4 Discussion

In this paper we have presented a novel approach to detect neonatal seizures based on a set of diverse features, which capture energy, frequency and structural nature of the EEG signal, and a statistical machine learning algorithm – SVM – to derive a data-driven non-linear function to discriminate between seizure and non-seizure. The system showed the best performance (up to this date) of a neonatal seizure detection system. The resultant methodology is patient-independent, that is, the model trained has not seen the testing patient beforehand. The patient-independent model produced is robust to a seizure-channel connection usually observed in patient-dependent seizure detectors which implies that the system does not expect a seizure to happen in a particular channel (Runarsson and Sigurdsson, 2005). Additionally, the post-processing steps make the system applicable to any number of EEG channels. This can be very beneficial in clinical practice as increasing the number of electrodes results in higher spatial localisation of seizures, while reducing the number of electrodes can reduce the setup time and patient handling time. We are currently investigating the behaviour and performance of the system with a reduced number of electrodes. Unlike many alternatives, which are based on a set of heuristic rules and thresholds, the probabilistic interpretation of the final decision allows for decision control by simple selection of confidence levels, which makes the proposed system flexible for clinical needs (Deburchgraeve et al., 2008; Navakatikyan et al., 2006).

In Temko et al. (2010) a number of different metrics for the system presented in this study are discussed and calculated. A more detailed analysis of the behaviour of this system is possible, based on these metrics. Moreover, the various ways for performance assessment used in the literature for reporting the performance of neonatal seizure detectors are outlined. Together, this allows a proper quantitative comparison of the proposed system to the leading alternatives. It is shown in (Temko et al., 2010) that reporting only the most commonly used event-based metrics such as GDR and FD/h can be misleading and insufficient for a complete evaluation. A comparison and thorough review of alternatives are not reported in this study but can be found in (Temko et al., 2010). Additionally, the reported epoch-based metrics (Temko et al., 2010) allow a better quantitative assessment of the detected seizure burden – a very important clinical measure which remains unrecognised when using conventional event-based GDR and FD/h metrics.

The future work will be devoted to addressing the limitations of the system. In particular, the most common sources and reasons for misclassification outlined in Sections 3.1 and 3.2 will be tackled and the solutions will be incorporated into the current system. We will also focus on increasing the robustness of the system which has become an important issue to be addressed as the designed system nears practical use in various application areas. Distortions introduced by a change in type of sensors/electrodes, a change of montage, mismatch of recording equipment, a difference in experience of clinical staff and the presence of various environment-specific non-biological artifacts can significantly affect the signal quality and consequently the system performance. For this, the proposed algorithm will be tested on a completely new, unlabelled, large dataset composed of recordings collected over various institutions and hospitals.

## 5 Conclusion

An SVM-based multi-channel neonatal seizure detection system has been presented. The system has been validated on a large clinical dataset of babies with seizures and the results have been reported. The proposed analysis of errors has overviewed common sources and reasons of misclassification with several examples shown. The proposed SVM-based seizure detection system allows for control of the final decision by choosing different confidence levels which makes the proposed system flexible for clinical needs. In future work we will focus on integrating the feedback outlined in the analysis of errors to further improve the robustness and performance of the neonatal seizure detector.

## Figures and Tables

**Fig. A1:**
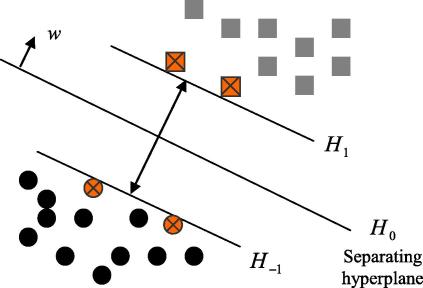
Two-class linear classification. The support vectors are indicated with crosses.

**Table 1:** EEG dataset.

Patient	Record length (h)	Seizure events	Mean seizure duration	Min seizure duration	Max seizure duration
1	18.23	17	1′30″	17″	3′54″
2	24.74	3	6′10″	55″	11′09″
3	24.24	149	2′18″	10″	10′43″
4	26.10	60	1′03″	25″	1′46″
5	24	49	5′54″	21″	31′01″
6	5.69	41	1′09″	26″	1′53″
7	24.04	6	1′04″	18″	1′28″
8	24.53	17	5′57″	29″	19′14″
9	24.04	156	5′16″	16″	37′06″
10	10.06	25	5′26″	10″	21′22″
11	6.19	15	5′26″	26″	7′49″
12	12	29	2′11″	13″	6′24″
13	12.13	25	4′06″	71″	12′16″
14	5.48	11	8′34″	69″	30′36″
15	12.16	59	2′05″	11″	7′08″
16	7.63	31	10′23″	2′14″	34′37″
17	6.64	12	8′32″	44″	23′16″

Total	267.9	705	–		

**Table 2:** Extracted features.

Groups	Feature list
Frequency domain	• Total power (0–12 Hz) • Peak frequency of spectrum • Spectral edge frequency (80%, 90%, 95%) • Power in 2 Hz width sub-bands (0–2 Hz, 1–3 Hz, …, 10–12 Hz) • Normalized power in sub-bands • Wavelet energy (the EEG is decomposed into eight coefficients using the Daubechy four wavelet, the energy in the fifth coefficient corresponding to 1–2 Hz is used as a feature)
Time domain	• Curve length • Number of maxima and minima • Root mean squared amplitude • Hjorth parameters • Zero crossings (raw epoch, Δ, ΔΔ) • Autoregressive modelling error (model order 1–9) • Skewness • Kurtosis • Non-linear energy • Variance (Δ, ΔΔ)
Information theory	• Shannon entropy • Singular value decomposition entropy • Fisher information • Spectral entropy

**Table 3:** Performance of the SVM-based seizure detection system for each patient.

Patient	GDR at 0.5 FD/h	GDR at 1 FD/h	Number of seizures
1	58.8	58.8	17
2	66.7	66.7	3
3	84.0	95.3	149
4	93.2	100	60
5	63.2	93.9	49
6	100	100	41
7	50	66.7	6
8	72.1	92.7	17
9	100	100	156
10	60	60	25
11	93.3	93.3	15
12	79.3	96.6	29
13	100	100	25
14	90	100	11
15	89.4	92.7	59
16	100	100	31
17	88.8	100	12

Mean	81.7	89.2	–

## Appendix A Construction of a SVM classifier

Let us assume a typical two-class problem in which the training patterns (vectors) $x_{i}\in R^{n}$ are linearly separable, as in Burges (1998), where the decision surface used to classify a pattern as belonging to one of the two classes is the hyperplane *H*_0_ (Fig. A1). If *x* is an arbitrary vector ($x\in R^{n}$), we define

(3) f(x)=w·x+b

where $w\in R^{n}$ and · denotes the dot product. *H*_0_ is the region of vectors *x* which verify the equation f(x)=0 (Scholkopf and Smola, 2002), and *H*_1_ and *H*_−1_ are two hyperplanes parallel to *H*_0_, and defined by *f*(*x*) = 1 and *f*(*x*) = −1, respectively. The distance separating the *H*_1_ and *H*_−1_ hyperplanes is

(4) $$d=\frac{2}{\left\|w\right\|}$$

and it is called the *margin*. The margin must be maximal in order to obtain a classifier that has not adapted too much to the training data, i.e. with good generalization characteristics. As we will see, the decision hyperplane *H*_0_ directly depends on vectors closest to the two parallel hyperplanes *H*_1_ and *H*_2_, which are called *support vectors*.

Consider a set of training data vectors $X=\{x_{1}\text{,}\ldots \text{,}x_{L}\}\text{,}\;x_{i}\in R^{n}$, and a set of corresponding labels $Y=\{y_{1}\text{,}\ldots \text{,}y_{L}\}\text{,}\;y_{i}\in \{1\text{,}\;-1\}$. We consider that the vectors are optimally separated by the hyperplane *H*_0_ if they are classified without error and the margin is maximal. In order to be correctly classified, the vectors must verify

(5) $${\mathrm{wx}}_{i}+b⩾+1\;\text{for}\;y_{i}=+1$$

(6) $${\mathrm{wx}}_{i}+b⩽-1\;\text{for}\;y_{i}=-1$$

Or, more concisely,

(7) $$y_{i}({\mathrm{wx}}_{i}+b)⩾1\text{,}\;\forall i$$

Thus the problem of finding the SVM classifying function *H*_0_ can be stated as follows:

(8) $$\text{minimize}\;\frac{1}{2}\|w{\|}^{2}$$

(9) $$\text{subject to}\;y_{i}({\mathrm{wx}}_{i}+b)⩾1\text{,}\;\forall i$$

Data observed in real conditions are frequently affected by outliers. Sometimes they are caused by noisy measurements. If the outliers are taken into account, the margin of separation decreases so the solution does not generalize so well, and the data patterns may no longer be linearly separable. To account for the presence of outliers, we can *soften* the decision boundaries by introducing a *slack* positive variable *ξ_i_* for each training vector. Thus, we can modify Eqs. (5) and (6) in the following way:

(10) $${\mathrm{wx}}_{i}+b⩾+1-\xi_{i}\;\text{for}\;y_{i}=+1$$

(11) $${\mathrm{wx}}_{i}+b⩽-1+\xi_{i}\;\text{for}\;y_{i}=-1$$

Obviously, if we take *ξ_i_* large enough, the constraints in Eqs. (10) and (11) will be met for all *i*. To avoid the trivial solution of large *ξ_i_*, we introduce a penalty cost in the objective function in Eq. (8), and thus the formulation becomes:

(12) $$\text{minimize}\;\left(\frac{1}{2}\|w{\|}^{2}+C\overset{L}{\underset{i=1}{\sum }}\xi_{i}\right)$$

(13) $$\text{subject to}\;y_{i}({\mathrm{wx}}_{i}+b)⩾1-\xi_{i}\text{,}\;\forall i$$

where *C* is a positive regularization constant which controls the degree of penalization of the slack variables *ξ_i,_* so that, when *C* increases, fewer training errors are permitted, though the generalization capacity may degrade. The resulting classifier is usually called a *soft margin classifier.* If *C *= ∞, no value for *ξ_i_* except 0 is allowed; it is the so-called *hard margin* SVM case.

The convex optimization problem in Eq. (12) with constraints in Eq. (13) is solved with Lagrangian multipliers *α_i_*. One of the most important features of SVM is that most patterns lie outside the margin area, and their optimal *α_i_*s are zero. Only those training patterns *x_i_* which lie on the margin surface or inside the margin area have non-zero *α_i_*, and they are named support vectors. Consequently, the classification process consists of assigning to any input vector *x* one of the two classes according to the sign of

(14) $$f(x)=\overset{M}{\underset{j=1}{\sum }}\alpha_{j}y_{j}x_{j}\cdot x+b$$

where *M* is the number of support vectors. The fact that the support vectors are a small part of the training data set makes the SVM implementation practical for large data sets.

For a non-linearly separable classification problem we have first to map the data onto a higher dimensional (possibly infinite) feature space where the data are linearly separable:

(15) $$f(x)=\overset{M}{\underset{j=1}{\sum }}\alpha_{j}y_{j}\phi (x)\cdot \phi (x_{j})+b$$

Notice the input vectors are involved in the expression through a dot product which can be substituted by a kernel function

(16) $$K(x_{i}\text{,}\;x_{j})=\phi (x_{i})\cdot \phi (x_{j})$$

The kernel can be thought as a non-linear similarity measure between two data points. Note that there is no need to know the mapping function ϕ explicitly, but only the kernel $K(x_{i}\text{,}\;x_{j})$.

The most often used kernel function in SVM applications is the Radial Basis Function (RBF):

(17) $$K(x_{i}\text{,}\;x_{j})=e^{-|x_{i}-x_{j}{|}^{2}/2\sigma }$$

Thus, from Eq. (15) and the kernel concept in Eq. (16), it follows that the two-class classification process with a SVM consists of assigning a positive/negative label to each input vector *x* through the following equation:

(18) $$y(x)=\text{sgn}\left(\overset{M}{\underset{j=1}{\sum }}\alpha_{j}y_{j}K(x\text{,}\;x_{j})+b\right)$$

where *M* is the number of support vectors.
